# Cytokine Profiling in Chinese SLE Patients: Correlations with Renal Dysfunction

**DOI:** 10.1155/2020/8146502

**Published:** 2020-10-09

**Authors:** Chen Yan, Le Yu, Xiu-Ling Zhang, Jing-Jing Shang, Jie Ren, Jie Fan, Xue-Qin Feng, Rong-Wei Zhang, Zhong-Bin Xia, Xin-Wang Duan

**Affiliations:** Department of Rheumatology, The Second Affiliated Hospital of Nanchang University, Nanchang 330006, China

## Abstract

**Background:**

Systemic lupus erythematosus (SLE) is a chronic, systemic autoimmune disease that commonly causes kidney damage. Therefore, we measured plasma levels of cytokines that may be related to renal dysfunction in SLE patients.

**Methods:**

To explore the differences between SLE patients with renal dysfunction and healthy volunteers, the levels of cytokines in plasma were screened using a human cytokine antibody array. Then, we chose fourteen of the elevated cytokines for verification with an expanded sample size by a human magnetic Luminex assay. Plasma samples were isolated from SLE patients (*n* = 72) and healthy volunteers (*n* = 8).

**Results:**

Cytokine antibody array data showed elevated plasma cytokines in SLE patients with renal dysfunction compared with healthy volunteers. By using the human magnetic Luminex assay, we found that plasma levels of CHI3L1, GDF-15, IGFBP-2, MIF, ST2, TFF3, and uPAR were significantly higher in SLE patients than in healthy volunteers. Plasma levels of CXCL4 were significantly lower in the active group than in the inactive group, and plasma levels of CHI3L1, IGFBP-2, MIF, and MPO were significantly higher in the active group than in the inactive group. We also analyzed the correlation between plasma cytokine levels and the SLEDAI-2K, and our results showed that the plasma levels of the fourteen selected cytokines were weakly correlated or not correlated with the SLEDAI-2K. We further analyzed the correlation between cytokines and renal dysfunction. Plasma levels of GDF-15 and TFF3 were highly positively correlated with serum creatinine levels and 24-hour urine protein levels.

**Conclusion:**

Our data suggest that plasma levels of GDF-15 and TFF3 are potential renal dysfunction markers in SLE patients, but plasma levels of these cytokines are not correlated with the SLEDAI-2K. Further study is warranted to determine how these cytokines regulate inflammatory responses and renal dysfunction in SLE.

## 1. Introduction

Systemic lupus erythematosus (SLE) is a chronic, systemic autoimmune disease that causes damage to multiple organ systems and is characterized by antinuclear antibody (ANA) production [1, 2]. The prevalence of SLE in the Chinese population was estimated to be approximately 40 cases per 100,000 persons [3]. Considering the huge population of China, this would be the largest cluster of cases in the world. During the last decade, there has been an increased understanding of the underlying autoimmune process in SLE, including the dysregulation of cytokines, increased expression of type I interferon- (IFN-) regulated genes, and activation of autoreactive B cells [2, 4]. It was also suggested that immune dysregulation precedes the development of clinical disease in SLE [4, 5].

Lupus nephritis (LN) significantly impacts the quality of life and longevity of SLE patients [6]. Renal dysfunction is the second leading cause of death in Chinese SLE patients [7]. However, traditional clinical parameters are not sensitive or specific enough to detect activity and early relapse of LN [8]. It has been reported that several cytokines in patients with SLE, such as IFN-*α*, B lymphocyte stimulator (BLyS), IL-17, and IL-1*β*, are related to disease activity and organ involvement [9–11]. To identify the potential biomarkers of LN, it is critical to know the roles of cytokines in renal dysfunction in SLE patients.

In this study, we investigated the correlation between plasma cytokine levels and renal dysfunction in Chinese SLE patients. We observed that plasma levels of GDF-15 and TFF3 are potential renal dysfunction markers in Chinese SLE patients. Furthermore, it should be noted that the expression and function of cytokines in SLE were significantly affected by different races/ethnicities and environmental exposures.

## 2. Materials and Methods

### 2.1. Patients and Samples

Patients with SLE (scoring ≥ 4 in the 1997 updated American College of Rheumatology (ACR) classification criteria) were recruited from the Second Affiliated Hospital of Nanchang University between 2017 and 2019. Seventy-two SLE patients and eight healthy volunteers were recruited. All patients provided informed written consent, and the study was approved by the Ethics Committee of the Second Affiliated Hospital of Nanchang University (No. 201620). Patients' disease activity and concurrent medication were assessed by a physician, and fasting blood samples were collected. Clinical features and serological data relevant to the ACR classification criteria were collected retrospectively from hospital records. Serum autoantibodies and complement levels were measured using standard laboratory assays. SLE disease activity was measured using the Systemic Lupus Erythematosus Disease Activity Index 2000 (SLEDAI-2K). Active disease was defined as SLEDAI‐2K > 4. All plasma samples were stored at -80°C until analysis.

### 2.2. Human XL Cytokine Array

To explore the differences in the levels of cytokines between SLE patients with renal dysfunction (*n* = 3, SLE group) and healthy volunteers (*n* = 3, control group), the average levels of cytokines in plasma were compared among the groups using a human XL cytokine Proteome Profiler™ array (ARY022b, R&D Systems, United States) per the manufacturer's instructions. Briefly, premixed plasmas were incubated with blocked membranes overnight at 4°C. The membranes were washed, incubated with a detection antibody cocktail for 1 hour, and washed three times, followed by incubation with streptavidin conjugated to horseradish peroxidase for 30 minutes. Expression was visualized using an enhanced chemiluminescence detection kit (#RPN2106, GE Healthcare Life Sciences, United States). Semiquantitative analysis was performed by measuring the density of the bands using an ImageQuant LAS 4000 mini biomolecular imager (GE Healthcare Life Sciences, United States).

### 2.3. Quantification of Plasma Cytokines

To detect the cytokine levels in plasma, we performed a human magnetic Luminex assay (LXSAHM-14) using a Luminex X-200. Fourteen cytokines were detected, including BLyS, C-C chemokine ligand 5 (CCL5), chitinase 3 like 1 (CHI3L1), C-X-C motif ligand 4 (CXCL4), growth differentiation factor 15 (GDF-15), intercellular cell adhesion molecule-1 (ICAM-1), insulin-like growth factor binding protein-2 (IGFBP-2), macrophage migration inhibitory factor (MIF), myeloperoxidase (MPO), resistin, serpin E1, suppression of tumorigenicity 2 (ST2), trefoil factor 3 (TFF3), and urokinase-type plasminogen activator receptor (uPAR). Briefly, 50 *μ*L of the standard or sample and 50 *μ*L of the microparticle cocktail were added to each well of the microplate. The plate was incubated for 2 hours at room temperature on a horizontal orbital microplate shaker. After the plate was washed, 50 *μ*L of the diluted biotin-antibody cocktail was added to each well and incubated for 1 hour at room temperature on a shaker. After the plate was washed, 50 *μ*L of diluted streptavidin-PE was added to each well. The plate was incubated for 30 minutes at room temperature on the shaker. After the plate was washed, the samples were read within 90 minutes using a Luminex X-200 analyzer.

### 2.4. Statistical Analyses

All data are presented as the mean ± SD. Whether the variables in each group were normally distributed was determined. To analyze the data that were not normally distributed, logarithmic transformation was performed. Statistical analysis was performed by the unpaired *t*-test between two groups, and correlations between variables were assessed using the Pearson test (GraphPad Prism 7, GraphPad Software, Inc.). A *p* value less than 0.05 was accepted as significant.

## 3. Results

### 3.1. Elevated Cytokine Levels in SLE Patients Compared with Healthy Volunteers

**Figure 1:**
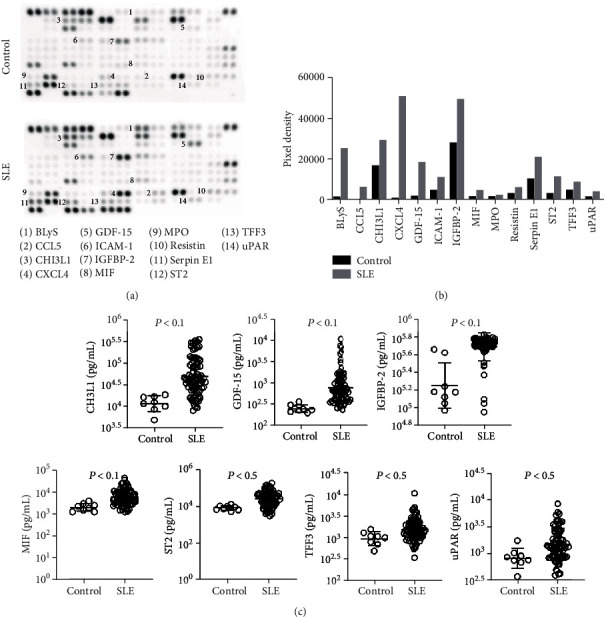
Elevated cytokine levels in SLE patients compared with healthy volunteers. (a) Raw images of the cytokine array. (b) Pixel densities of the fourteen selected cytokines. (c) Quantitative analysis of the level of cytokines in SLE patients and healthy volunteers.

Three SLE patients and three healthy volunteers were recruited for the human XL cytokine Proteome Profiler™ array, of whom 3/3 (100%) and 3/3 (100.0%) were female, with mean (±SD) ages of 34.00 ± 7.55 and 29.33 ± 9.24 years, respectively (Table 1). Cytokine array data showed elevated cytokine levels in the plasma of SLE patients compared with healthy volunteers (Figure 1(a)). Then, we chose fourteen of the elevated cytokines for verification with an expanded sample size (Figure 1(b)).

Seventy-two SLE patients and eight healthy volunteers were recruited, of whom 65/72 (90.3%) and 8/8 (100.0%) were female, with mean (±SD) ages of 32.83 ± 10.53 and 31 ± 6.44 years, respectively (Table 2). Plasma levels of CHI3L1, GDF-15, IGFBP-2, MIF, ST2, TFF3, and uPAR were significantly higher in SLE patients compared with healthy volunteers (Figure 1(c)). There were no significant differences in the other 7 cytokines (Supplementary Fig. 1).

### 3.2. Differential Expression of Cytokines in Active and Inactive SLE Patients

**Figure 2:**
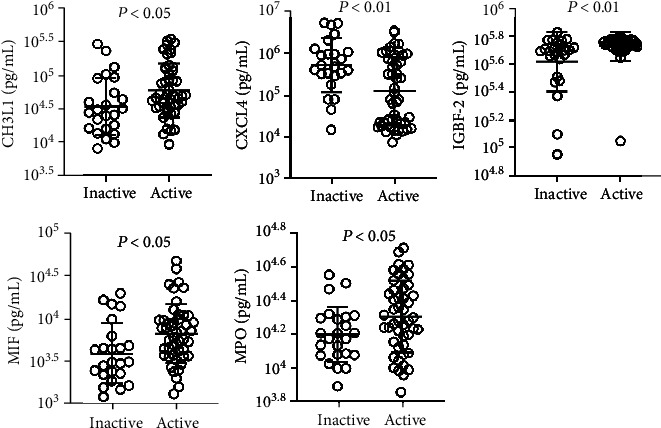
Differential expression of cytokines in inactive and active SLE patients.

**Figure 3:**
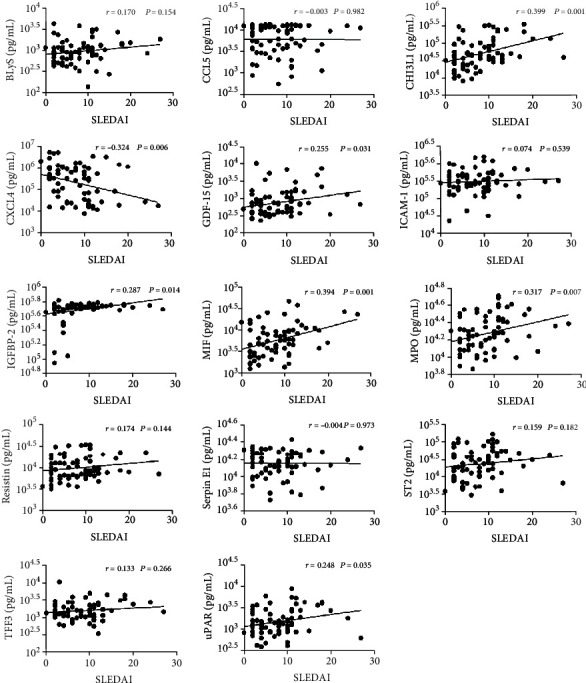
Correlation of plasma cytokine levels with clinical parameters of disease activity.

In the SLE patient group, 48/72 patients (66.7%) had active disease (SLEDAI‐2K > 4). The active group was younger than the inactive group (32.04 ± 10.52 vs. 34.42 ± 10.58 years) (Table 2). Plasma levels of CXCL4 were significantly lower in the active group than in the inactive group, and plasma levels of CHI3L1, IGFBP-2, MIF, and MPO were significantly higher in the active group compared with the inactive group (Figure 2). There were no significant differences in the other 9 cytokines (Supplementary Fig. 2). We also analyzed the correlation between plasma cytokine levels and the SLEDAI-2K, and our results showed that the plasma levels of these cytokines were weakly correlated or uncorrelated with the SLEDAI-2K (Figure 3).

### 3.3. Correlation of Plasma Cytokine Levels with Clinical Parameters of Renal Dysfunction

**Figure 4:**
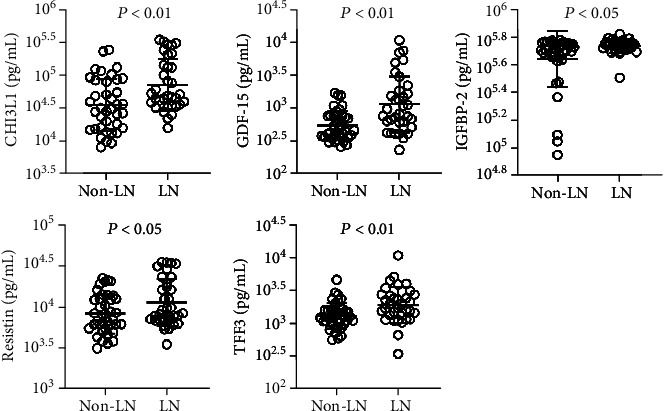
Differential expression of cytokines in LN and non-LN patients.

**Figure 5:**
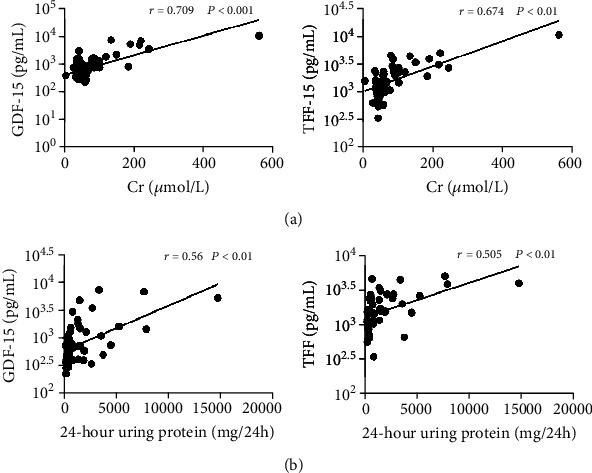
Correlation of plasma cytokine levels with clinical parameters of renal dysfunction. (a) Correlation between plasma cytokine levels and serum creatinine levels. (b) Correlation between plasma cytokine levels and 24-hour urine protein levels.

We further analyzed the correlation between cytokines and renal dysfunction. Plasma levels of CHI3L1, GDF-15, IGFBP-2, resistin, and TFF3 were significantly higher in LN patients than in SLE patients without renal involvement (Figure 4). There were no significant differences in the other 9 cytokines (Supplementary Fig. 3). Plasma levels of GDF-15 and TFF3 were highly positively correlated with serum creatinine levels and 24-hour urine protein levels (*r* > 0.5, *p* < 0.01, Figures 5(a) and 5(b)). There was no obvious correlation between renal dysfunction and the other 11 cytokines (Supplementary Fig. 4 and Supplementary Fig. 5).

## 4. Discussion

Cytokine dysregulation is a characteristic of SLE [2, 12]. Increased plasma cytokine levels have been found in SLE patients. These cytokines, in turn, have a number of effects that drive lupus pathophysiology, as well as the increased organ damage. In our study, plasma levels of CHI3L1, GDF-15, IGFBP-2, MIF, ST2, TFF3, and uPAR were significantly higher in SLE patients than in healthy volunteers. Consistent with our study, it has been reported that plasma levels of IGFBP-2, MIF, ST2, and uPAR were increased in SLE patients compared with healthy volunteers [13–19]. Previous studies suggested MIF as a therapeutic target for SLE, as *in vivo* miRNA inhibition of MIF decreased downstream cytokine production and ameliorated murine lupus nephritis [13, 14]. However, the role of elevated plasma CHI3L1, GDF-15, and TFF3 in SLE patients is still unknown.

Moreover, it has been reported that the disease activity of SLE is related to cytokine levels, such as pentraxin-related protein (PTX3) and C-X-C motif ligand 10 (CXCL10) [9]. We found decreased CXCL4 levels and elevated CHI3L1, IGFBP-2, MIF, and MPO levels in active SLE patients compared with inactive SLE patients. In addition, plasma levels of these cytokines were weakly or not correlated with the SLEDAI-2K. It has been reported that serum IGFBP-2 levels were significantly higher in patients with active SLE than in those with inactive SLE or in healthy volunteers in a cohort of Chinese patients with SLE [17]. Increased plasma MPO levels in SLE have also been reported in Brazil [20]. However, there was no significant difference in plasma MPO levels between active SLE patients and inactive SLE patients, and there was no correlation between plasma MPO levels and SLEDAI-2K (*r* = 0.07, *p* = 0.58) [20]. A previous study from Australia also reported decreased serum MPO levels in SLE patients compared with healthy volunteers [21]. However, we found higher plasma MPO levels in active SLE patients than in inactive SLE patients, and plasma MPO levels were weakly correlated with the SLEDAI-2K (*r* = 0.317, *p* = 0.007). In contrast, a study in Greece showed that plasma levels of CXCL4 were comparable between SLE patients and healthy volunteers [22]. It is not clear why the research results in different countries are inconsistent. It seems that environmental exposure and race/ethnicity may affect the expression and function of cytokines in SLE [23, 24].

Most organs can be involved in SLE, and the typical major organ manifestations are in the kidney and central nervous system [25]. It has been found that more lupus nephritis cases and fewer neuropsychiatric lupus cases were detected in Chinese patients with SLE than in SLE patients from other countries [26]. A higher percentage of Chinese patients presented with nephropathy (47.4%) than European patients (27.9%) [26]. It is necessary to pay close attention to the diagnosis and treatment of renal dysfunction in Chinese SLE patients. Novel serum and urinary biomarkers, such as cytokines and chemokines, have been evaluated for detecting early flares in LN [8]. In our results, plasma levels of CHI3L1, GDF-15, IGFBP-2, resistin, and TFF3 were significantly higher in LN patients than in SLE patients without renal involvement. Moreover, plasma levels of GDF-15 and TFF3 were highly positively correlated with clinical parameters of renal dysfunction. IGFBP-2 may differentiate active renal SLE from active nonrenal or inactive SLE [17]. However, our results showed that the level of IGFBP-2 was not correlated with clinical parameters of renal dysfunction (*p* > 0.05, Supplementary Fig. 3 and Supplementary Fig. 4). It has been reported that GDF-15 and TFF3 are related to renal dysfunction diseases. GDF-15 is suggested to be a marker of cardiac injury and renal dysfunction in patients undergoing cardiac surgery [27, 28]. Circulating GDF-15 levels are significantly associated with an increased risk of CKD progression [29]. TFF3 has been suggested to be a highly sensitive and specific urinary biomarker to monitor drug-induced kidney injury in clinical trials [30]. In addition, serum and urine concentrations of TFF3 were correlated with the stage of CKD severity and these increased concentrations of TFF3 may be due to secretion from renal tubular epithelial cells in damaged kidneys [31]. Although the roles of GDF-15 and TFF3 in SLE were not clear, our results suggest that the plasma levels of GDF-15 and TFF3 reflect the severity of renal dysfunction in SLE.

In conclusion, our data suggest that plasma levels of GDF-15 and TFF3 are potential renal dysfunction markers in SLE patients, but plasma levels of these cytokines were weakly or not correlated with the SLEDAI-2K. Furthermore, it should be noted that the expression and function of cytokines in SLE were significantly affected by different races/ethnicities and environmental exposures. This study is largely limited by the relatively small sample size, and we did not investigate the relevant mechanisms of these cytokines in regulating SLE disease progression. Further study is warranted to expand the sample size and determine how these cytokines regulate inflammatory responses and renal dysfunction in SLE.

## Figures and Tables

**Table 1:** Characteristics of the study participants in the human XL cytokine array.

Characteristics	SLE patient with renal dysfunction (*n* = 3)	Healthy volunteers (*n* = 3)
Age (±SD) (years)	34.00 ± 7.55	29.33 ± 9.24
Gender (female)	3	3
Disease duration (±SD) (months)	22.67 ± 22.03	—
SLEDAI-2K (±SD)	6.67 ± 4.16	—
Cr (±SD) (*μ*mol/L)	185.7 ± 33.16	—
24-hour urine protein (±SD) (mg/24 h)	2933 ± 1955	—

**Table 2:** Characteristics of the study participants.

Characteristics	SLE (*n* = 72)	Healthy volunteers (*n* = 8)	Active SLE (*n* = 48)	Inactive SLE (*n* = 24)	LN (*n* = 34)	Non-LN (*n* = 38)
Age (±SD) (years)	32.83 ± 10.53	31 ± 6.44	32.04 ± 10.52	34.42 ± 10.58	32.26 ± 10.80	33.34 ± 10.40
Gender (female)	65	8	44	21	30	35
Disease duration (±SD) (months)	25.4 ± 44.22	—	18.31 ± 38.72	39.58 ± 51.55	24.11 ± 47.81	26.56 ± 41.35
SLEDAI-2K (±SD)	8.042 ± 5.476	—	10.75 ± 4.72	2.625 ± 1.056	10.82 ± 5.82	5.55 ± 3.73
Cr (±SD) (*μ*mol/L)	77.98 ± 74.31	—	78.55 ± 53.99	76.86 ± 104.8	105.9 ± 99.60	52.33 ± 15.24
24-hour urine protein (±SD) (mg/24 h)	1511 ± 2626	—	1875 ± 2846	126.7 ± 136.2	2514 ± 3045	138.7 ± 118.5

## Data Availability

The datasets generated and analyzed for the present study are available from the corresponding author on reasonable request.

## Supplementary Materials

Supplementary Materials Supplementary Fig. 1: quantitative analysis of the level of cytokines in SLE patients and healthy volunteers. Supplementary Fig. 2: differential expression of cytokines in the inactive and active SLE patients. Supplementary Fig. 3: differential expression of cytokines in LN patients compared with non-LN patients. Supplementary Fig. 4: correlation between plasma cytokine levels and serum creatinine levels. Supplementary Fig. 5: correlation between plasma cytokine levels and 24-hour urine protein levels.
