# Supplementary material for: Cytokine Profiling in Chinese SLE Patients: Correlations with Renal Dysfunction
Source: J Immunol Res. 2020 Oct 9;2020:8146502. doi: 10.1155/2020/8146502 (PMC7568803; doi:10.1155/2020/8146502)
Supplement: Supplementary Materials — Supplementary Fig. 1: quantitative analysis of the level of cytokines in SLE patients and healthy volunteers. Supplementary Fig. 2: differential expression of cytokines in the inactive and active SLE patients. Supplementary Fig. 3: differential expression of cytokines in LN patients compared with non-LN patients. Supplementary Fig. 4: correlation between plasma cytokine levels and serum creatinine levels. Supplementary Fig. 5: correlation between plasma cytokine levels and 24-hour urine protein levels. [file 8146502.f1.docx]

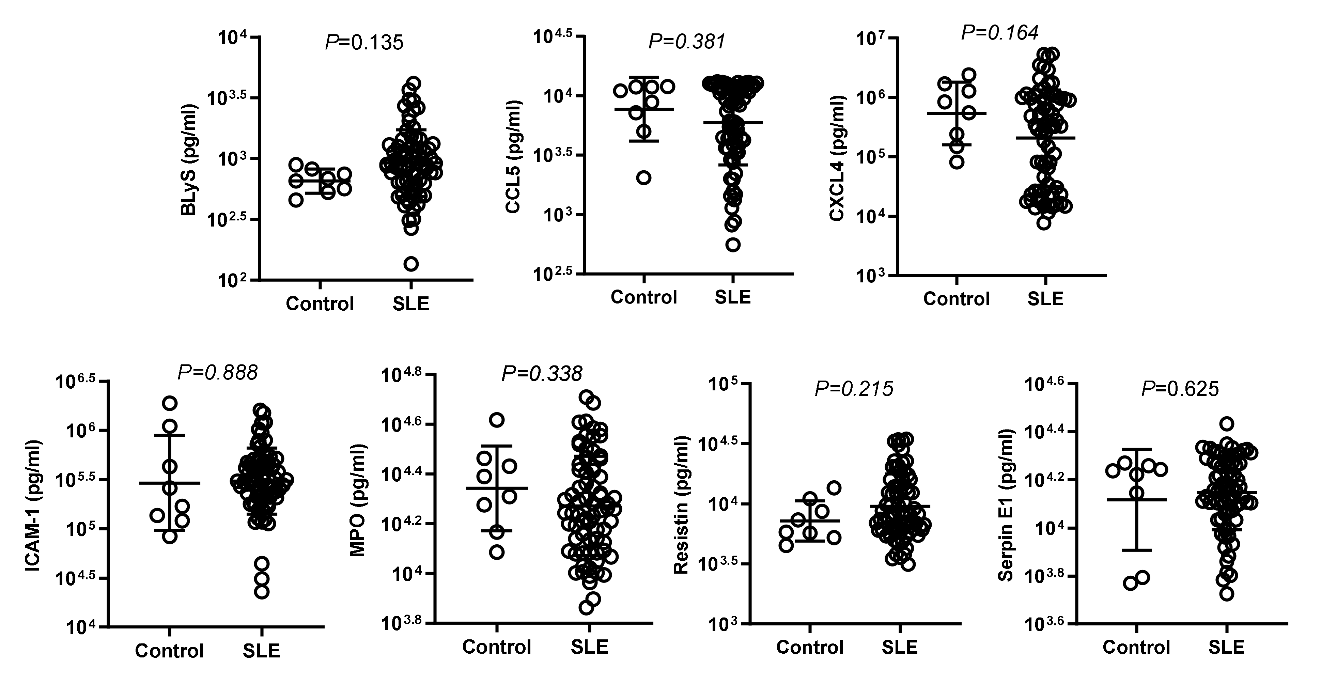


**Supplementary Fig. 1.** Quantitative analysis of the level of cytokines in SLE patients and healthy volunteers.


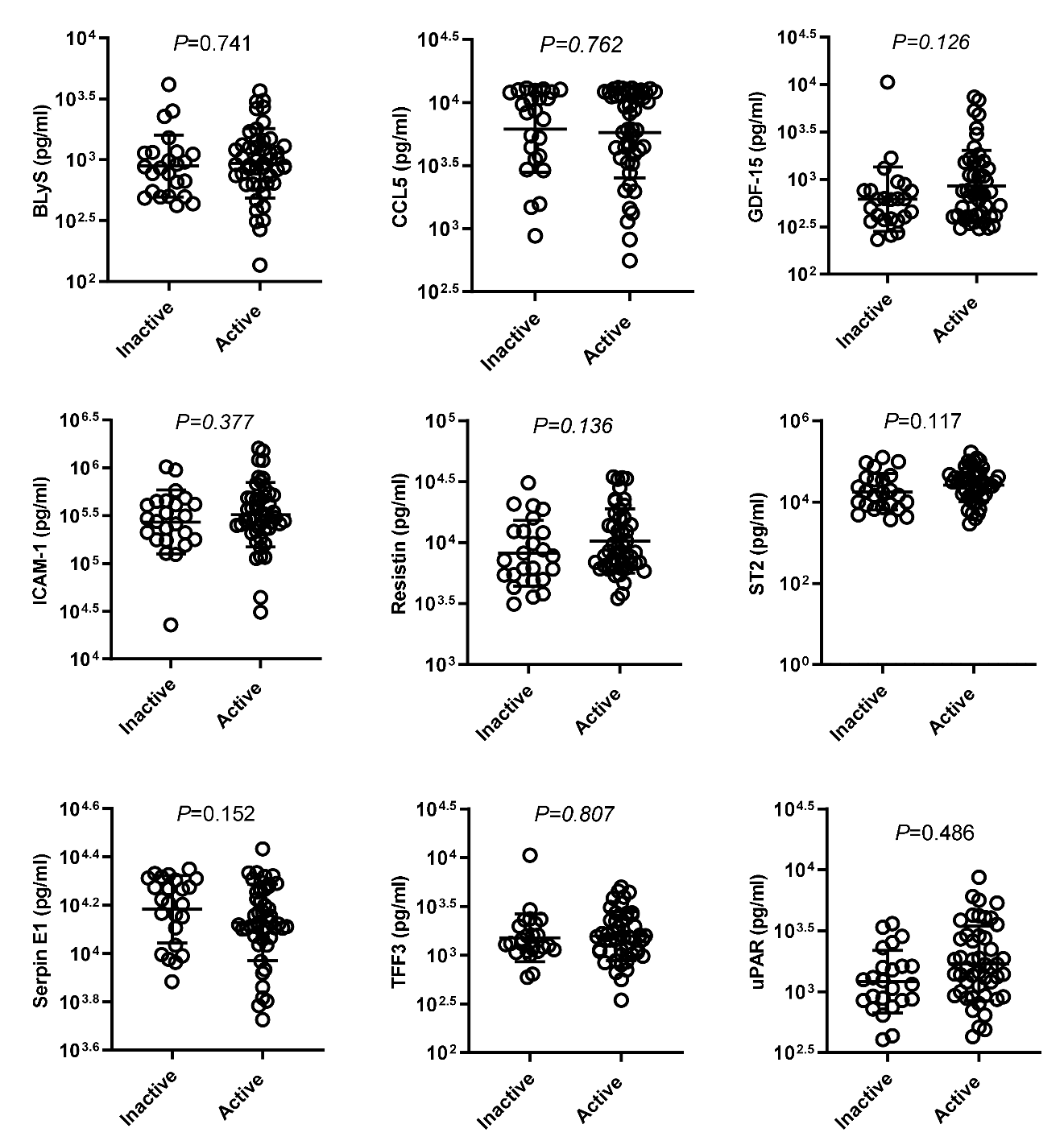


**Supplementary Fig. 2**. Differential expression of cytokines in the inactive and active SLE patients.


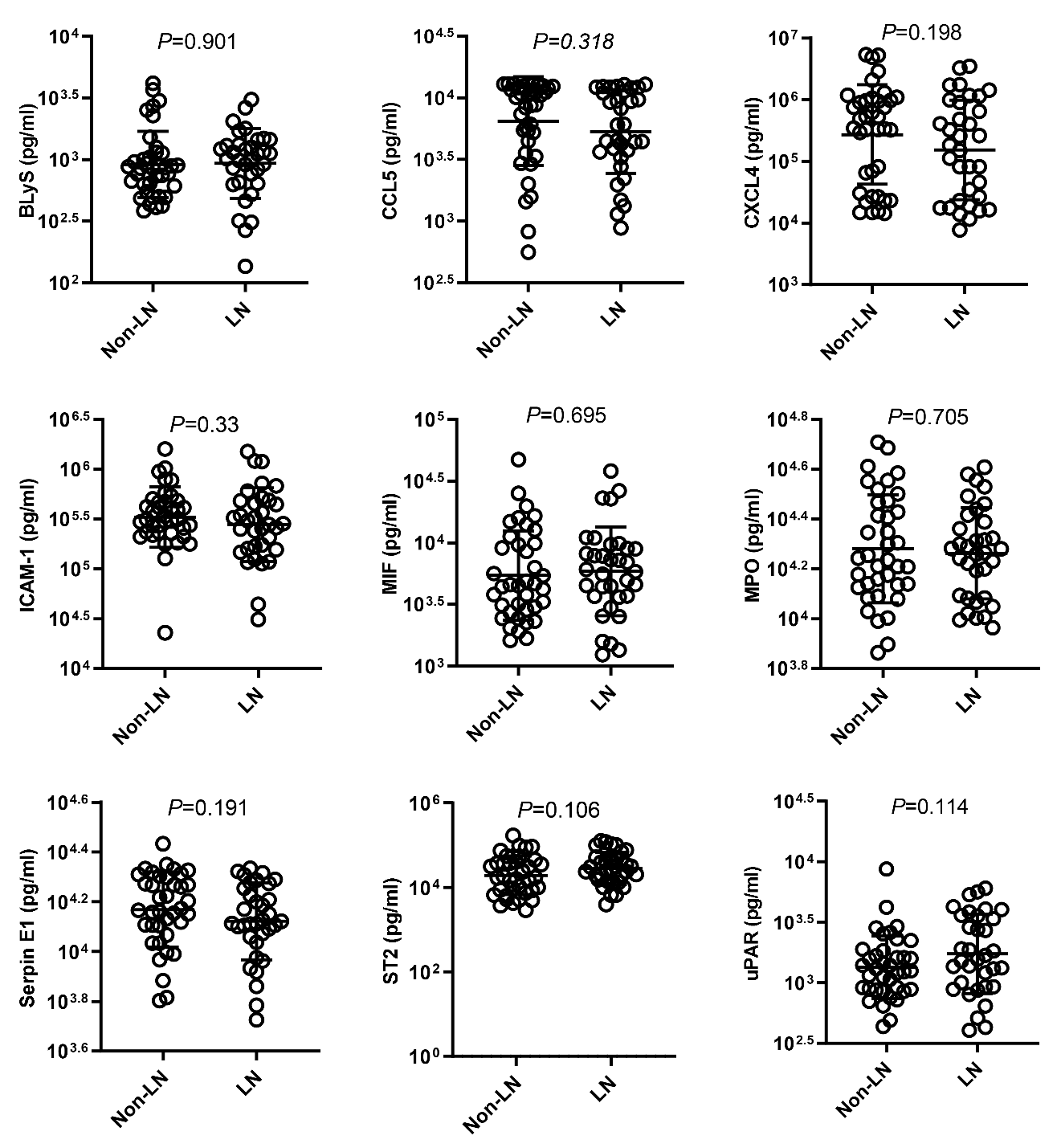


**Supplementary Fig. 3.** Differential expression of cytokines in LN patients compared with non-LN patients.


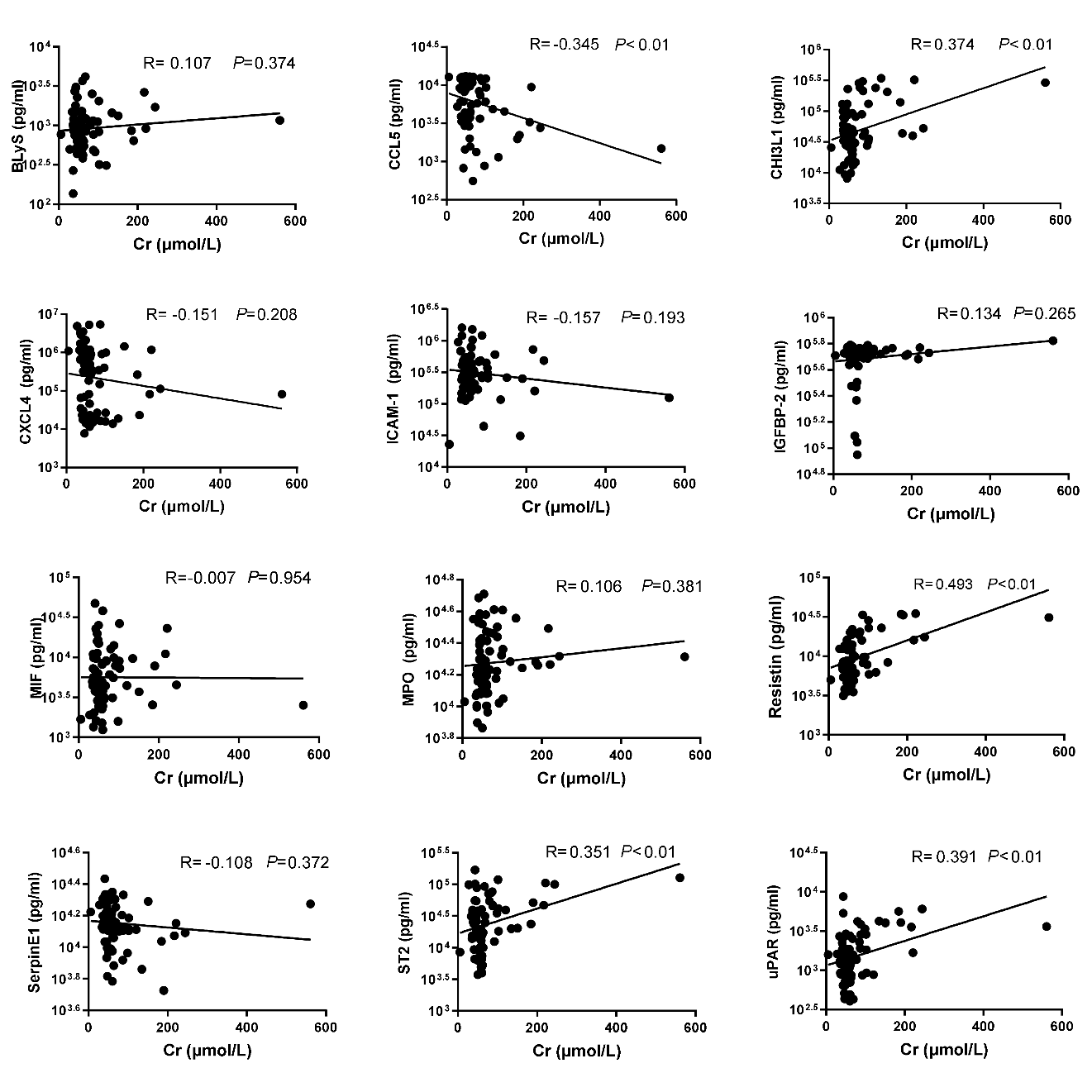


**Supplementary Fig. 4.** Correlation between plasma cytokine levels and serum creatinine levels.


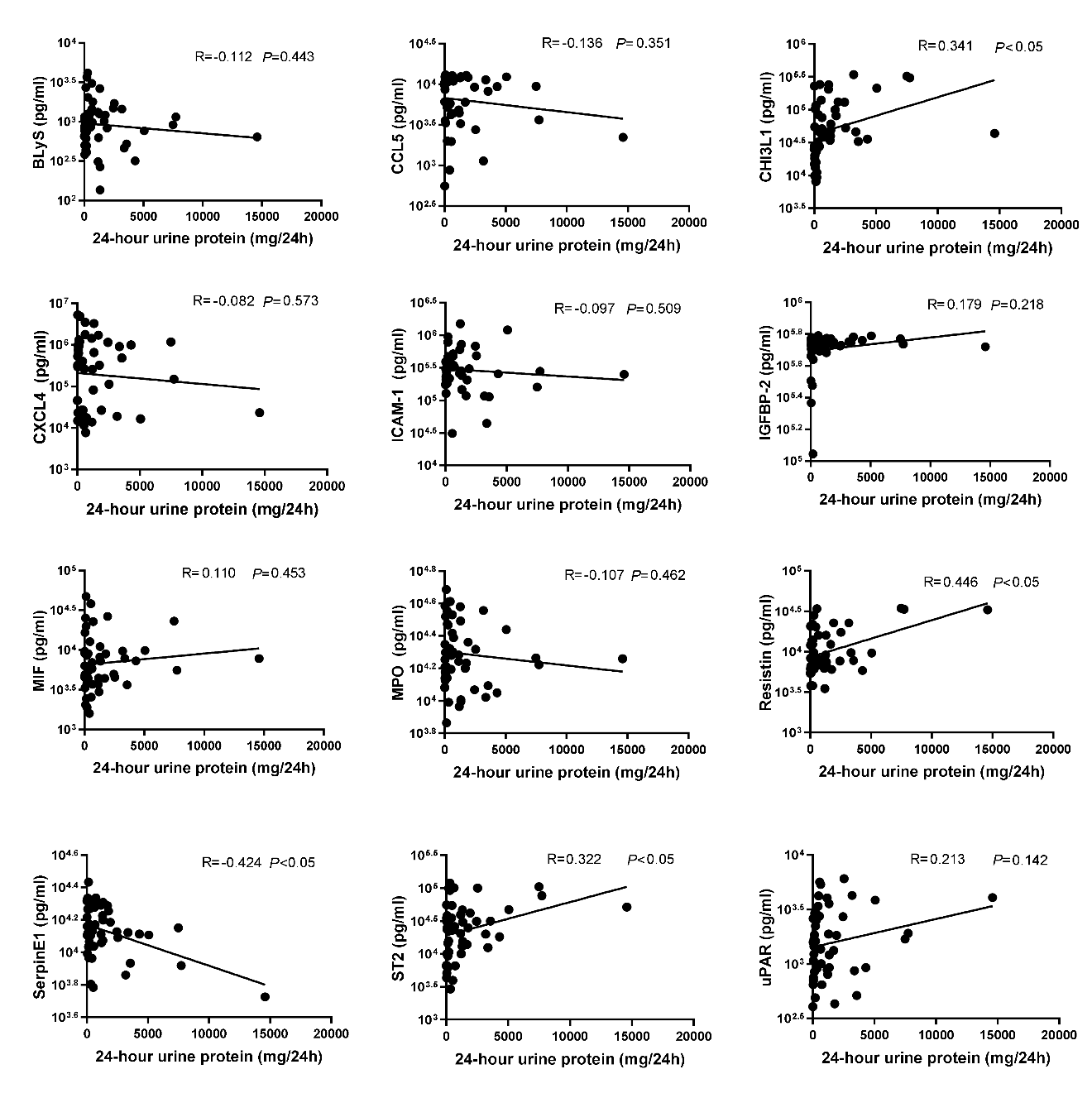


**Supplementary Fig. 5**. Correlation between plasma cytokine levels and 24-hour urine protein levels.
